# Placement of the Internal Pulse Generator for Deep Brain Stimulation in the Upper Back to Prevent Fracture of the Extension Wire due to Generator Rotation: Case Report

**DOI:** 10.4061/2010/189371

**Published:** 2010-02-08

**Authors:** Ankur Garg, Avinash L. Mohan, P. Charles Garell

**Affiliations:** Department of Neurosurgery, New York Medical College, Valhalla, NY 10595, USA

## Abstract

Deep brain stimulation (DBS) is a common surgical procedure used for the treatment of Parkinson's disease (PD) and essential tremor. A potential complication of this procedure is hardware failure. The authors report a case of DBS hardware failure in which repeated fractures of the extension wire were caused by abnormal rotational movements of the IPG placed in the loose subclavicular tissue of an overweight female. Implantation of the IPG in the suprascapular area prevented further extension wire fractures. This strategy may be especially relevant in overweight females with loose subclavicular tissue.

## 1. Introduction

Deep brain stimulation (DBS) is a common surgical procedure used for the treatment of Parkinson's disease (PD) and essential tremor. A potential complication of this procedure is hardware failure, which could be in the form of an electrode or extension lead fracture [1]. With the recent increase in the number of DBS surgeries, we are likely to see more cases of DBS hardware failure. Few reports of DBS hardware failure are currently in the neuroscience literature [1–5]. Such reports are important as they provide a basis not only for future improvements in the hardware technology but also for early diagnosis and appropriate management of similar events. Here we report on a patient who underwent bilateral subthalamic nucleus (STN) DBS implantation for PD and experienced three events of right-sided extension wire fractures. The fractures were presumably caused by mechanical strain on the extension wire due to rotation of the internal pulse generator (IPG) in a loose subclavicular pocket in an overweight female patient. Implantation of the IPG in the upper back led to successful treatment and prevented further extension wire fractures.

## 2. Case Report

Patient is a seventy-year-old right-handed female with eight-year history of PD. She was referred for DBS surgery in 2004 as she was having significant on/off effect and dyskinetic movements. 

### 2.1. Initial Operation

Bilateral STN-DBS surgery with two Soletra (Medtronic) units was performed in April 2004. The stimulation parameters were gradually adjusted, and medications were reduced to achieve optimal symptomatic improvement. Her dyskinesias and on/off effects were reduced significantly, and her swallowing and speech improved.

### 2.2. Second Operation

**Figure 1:**
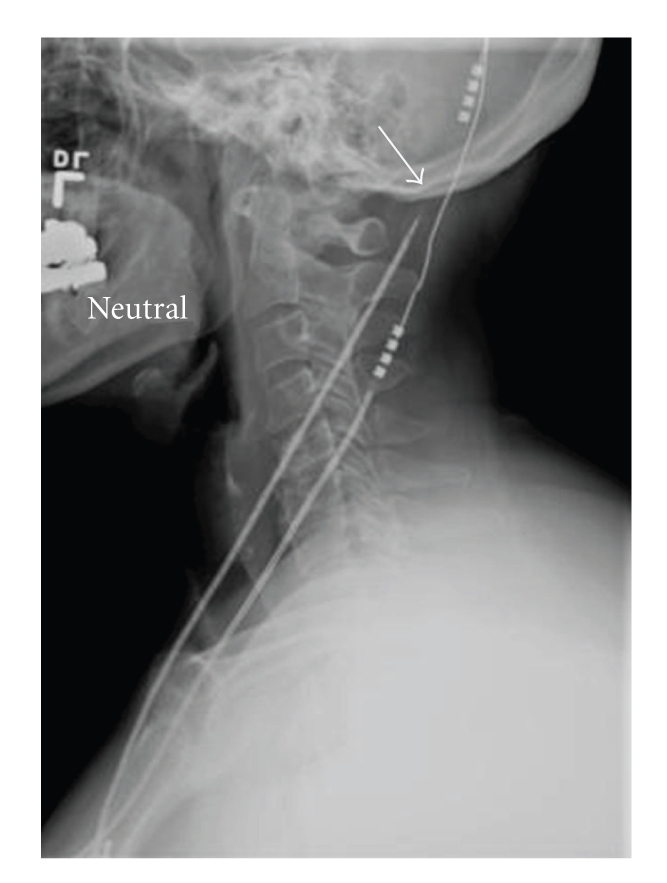
Lateral X-ray showing discontinuity of the right extension lead (arrow). Note that both the right and the left connections are low relative to the skull.

In April 2006, the patient reported that her symptoms were increasingly less well controlled and that she experienced occasional tingling-type sensations in the right retroauricular region. Interrogation of her DBS system revealed very high impedance (>2000 ohms) on all combinations on the right side, whereas impedance was within the normal range on the left side. An X-ray revealed a discontinuity in the integrity of the extension wire on the right (Figure 1). Elective replacement of the right-sided extension lead and IPG and movement of the connection on the skull surface were performed in May 2006. The patient reported immediate improvement in her symptoms. Postoperative interrogation of the DBS system revealed the impedance to be less than 1800 ohms on both sides. 

### 2.3. Third Operation

**Figure 2:**
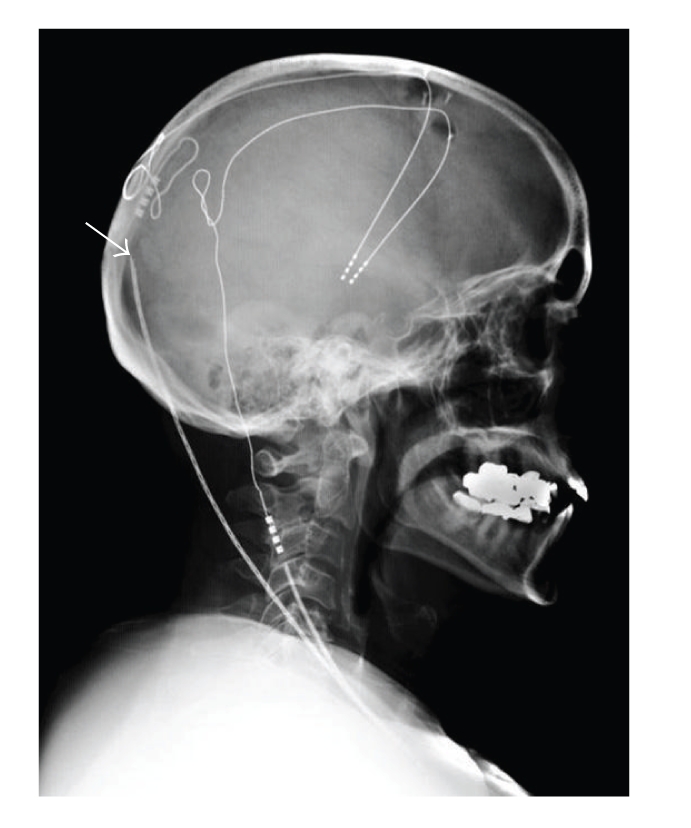
Lateral X-ray showing a new discontinuity of the right extension lead over the skull at the connection to the right DBS electrode. Note the braided appearance of the right extension lead.

**Figure 3:**
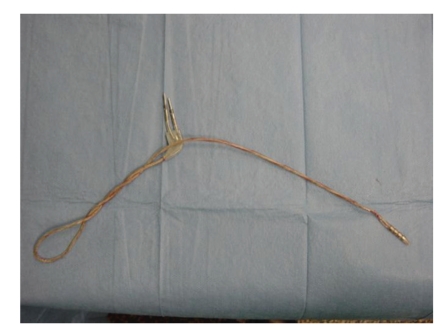
Appearance of the right extension lead during the third operation.

In August 2006, the patient reported increased bradykinesia and speech difficulties. Her medications were increased, resulting in mild to moderate dyskinesias. Interrogation of the DBS system revealed that the impedance on the right was again >2000 ohms for all combinations, whereas it remained <1800 ohms on the left side. Followup X-rays revealed a braided appearance and a new discontinuity of the right extension lead (Figure 2). On the basis of the clinical, electrophysiological, and radiological evidences, elective replacement of the right-sided extension lead was again performed in October 2006. At operation, the extension lead was extensively coiled on itself, presumably due to the rotation of the IPG in the loose connective tissue of the right subclavicular region (Figure 3). These rotational movements created an increasing mechanical strain on the extension lead, resulting eventually in its fracture at the more firmly attached connection site. To prevent recurrence, we created a smaller subclavicular pocket by obliterating the excessive dead space and sutured the IPG to the surrounding fascia through two suture holes in the IPG. The patient reported immediate improvement in her parkinsonian symptoms postoperatively.

### 2.4. Fourth Operation

**Figure 4:**
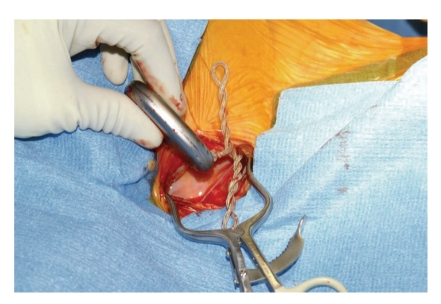
Appearance of the right IPG and extension lead during the fourth operation. Note the extensively coiled extension lead.

A followup X-ray in January 2007 revealed increased density in the right extension wire at multiple places that suggested new coiling. However, on interrogation, the DBS system was found to be electrically connected and functional. Followup X-rays in February 2007 revealed further increased coiling of the right extension wire. On interrogation of the right DBS system, the impedance was found to be high for all electrode combinations. At this point, we recommended placing the right IPG in the right suprascapular position. At revision surgery in May 2007, the IPG and extension lead were found to be extensively rotated (Figure 4). Postoperatively, the patient reported immediate improvement in her symptoms.

### 2.5. Followup

**Figure 5:**
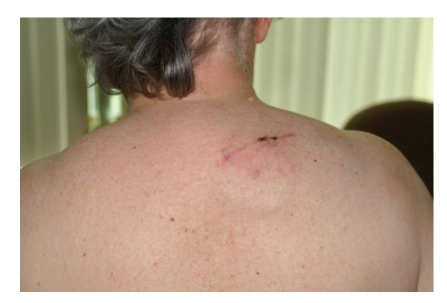
Postoperative appearance of the IPG implanted in the suprascapular region after the last surgery.

**Figure 6:**
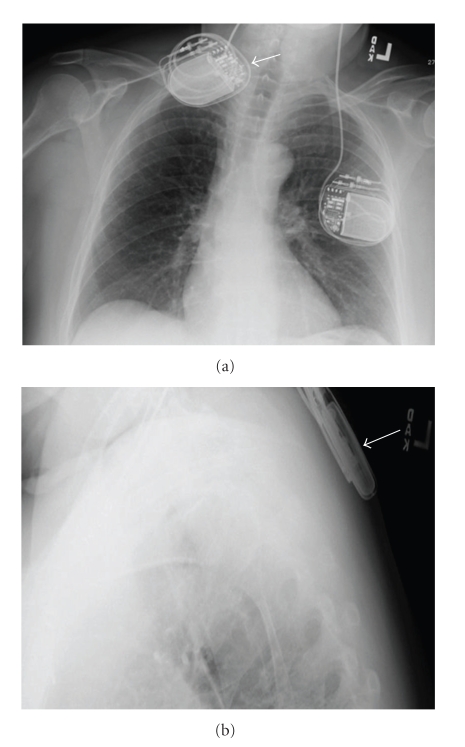
Postoperative X-ray showing placement of the right IPG in suprascapular region after the last surgery.

The patient has been followed for 12 months after the surgery and reports continued resolution of her symptoms (Figures 5 and 6). On interrogation on followup, the impedance was found to be within the normal range on both sides.

## 3. Discussion

DBS is now a well-established treatment modality for medically uncontrolled PD. As with any technology, hardware failures may occur with DBS and lead to recurrence of symptoms [1–5]. In a recent study [1] of 81 consecutive patients undergoing STN-DBS for PD, an incidence of 26.2% was found for hardware complications including lead migration, lead fracture, extension erosion, extension fracture, and IPG malfunction. Sudden deterioration of the patient's neurological condition and parkinsonian symptoms after DBS hardware failure have been reported [2, 3]. In a series of four IPG hardware failures, sudden and severe deterioration of the patient's neurological condition occurred [2]. In another series [3], two patients with advanced PD treated with bilateral DBS were described in whom unilateral battery depletion resulted in rapid appearance of disabling PD symptoms that did not respond to high doses of dopaminergic medications. In both series, the patients were restored to their previous level of function only and immediately after replacement of the device. 

 Our case is an unusual example of DBS hardware failure in which fractures of the extension wire were caused by abnormal rotational movements of the IPG placed in the loose subclavicular tissue of an overweight female. These rotations of the IPG created a continuous mechanical strain on the extension lead resulting in recurrent fractures. To prevent further recurrence, the following options were contemplated: leaving the right DBS “OFF” and continuing with the left DBS and medications, revision with the Kinetra IPG, revision with right IPG implantation in the abdomen, and revision with right IPG implantation in the right suprascapular position. Another option was to again place the IPG in the subclavicular region but this time more deeply and securely sutured to the subclavicular fascia; however, that would have placed the IPG too deep, given the patient's body habitus, and would not communicate with the programmer. We recommended IPG implantation in the suprascapular position because of the patient's concerns with the Kinetra IPG and our concern of a recurrence with implantation in the abdomen. Placement of IPGs in the abdomen has been reported [6], but we did not advocate this strategy for our patient because she had loose abdominal muscles that might have posed the same problem as her loose subclavicular tissue. The suprascapular location, by contrast, is a tight space with less potential for abnormal movement of the implanted material. This strategy proved to be effective for our patient in preventing further recurrence of IPG rotation and extension wire fracture.

## 4. Conclusions

With the recent increase in the number of DBS surgeries for PD and other neurological disorders, the number of patients presenting with DBS hardware problems will increase. Some of these hardware failures may require different strategies to prevent further episodes of recurrence. In the case of our patient, placement of the IPG in the suprascapular region provided an effective solution for preventing fracture of the extension wire caused by abnormal rotational movements of the IPG. This strategy may be especially relevant in overweight females with loose subclavicular tissue.
